# Naturally Acquired Human Immunity to Pneumococcus Is Dependent on Antibody to Protein Antigens

**DOI:** 10.1371/journal.ppat.1006137

**Published:** 2017-01-30

**Authors:** Robert Wilson, Jonathan M. Cohen, Mark Reglinski, Ricardo J. Jose, Win Yan Chan, Helina Marshall, Corné de Vogel, Stephen Gordon, David Goldblatt, Fernanda C. Petersen, Helen Baxendale, Jeremy S. Brown

**Affiliations:** 1 Centre for Inflammation and Tissue Repair, Division of Medicine, University College Medical School, Rayne Institute, London, United Kingdom; 2 Infectious Diseases & Microbiology Unit, UCL Institute of Child Health, London, United Kingdom; 3 Medical Microbiology and Infectious Diseases, Erasmus MC, Rotterdam, The Netherlands; 4 Respiratory Infection Group, Liverpool School of Tropical Medicine, Liverpool, United Kingdom; 5 Institute of Child Health, University College London, London, United Kingdom; 6 Department of Oral Biology, Faculty of Dentistry, University of Oslo, Norway; 7 Clinical Immunology Department, Papworth Hospital NHS Foundation Trust, Cambridge, United Kingdom; University of Birmingham, UNITED KINGDOM

## Abstract

Naturally acquired immunity against invasive pneumococcal disease (IPD) is thought to be dependent on anti-capsular antibody. However nasopharyngeal colonisation by *Streptococcus pneumoniae* also induces antibody to protein antigens that could be protective. We have used human intravenous immunoglobulin preparation (IVIG), representing natural IgG responses to *S*. *pneumoniae*, to identify the classes of antigens that are functionally relevant for immunity to IPD. IgG in IVIG recognised capsular antigen and multiple *S*. *pneumoniae* protein antigens, with highly conserved patterns between different geographical sources of pooled human IgG. Incubation of *S*. *pneumoniae* in IVIG resulted in IgG binding to the bacteria, formation of bacterial aggregates, and enhanced phagocytosis even for unencapsulated *S*. *pneumoniae* strains, demonstrating the capsule was unlikely to be the dominant protective antigen. IgG binding to *S*. *pneumoniae* incubated in IVIG was reduced after partial chemical or genetic removal of bacterial surface proteins, and increased against a *Streptococcus mitis* strain expressing the *S*. *pneumoniae* protein PspC. In contrast, depletion of type-specific capsular antibody from IVIG did not affect IgG binding, opsonophagocytosis, or protection by passive vaccination against IPD in murine models. These results demonstrate that naturally acquired protection against IPD largely depends on antibody to protein antigens rather than the capsule.

## Author Summary

*Streptococcus pneumoniae* is a major global killer. Invasive pneumococcal disease (IPD) is the most severe form of infection. Surprisingly, the natural mechanisms of immunity to IPD in healthy individuals are unclear. The success of vaccines stimulating anti-capsular antibodies have led to the belief that the same mechanism lies behind natural protection. Using studies with pooled human immunoglobulin, we demonstrate that this is not the case and instead IgG recognising the bacterial surface proteins appears to have the dominant functional role. This finding supports efforts towards protein antigen-based vaccines, and opens the possibility of stratifying potential risk for individuals of IPD.

## Introduction

*Streptococcus pneumoniae* is a leading cause of infectious disease related death, responsible annually for up to a million child deaths worldwide [1]. Pneumonia represents the greatest burden of disease caused by *S*. *pneumoniae* [2], and despite current vaccination strategies the burden of pneumococcal pneumonia remains high. Invasive pneumococcal disease (IPD) is the most severe form of *S*. *pneumoniae* infection and mainly affects very young children and older adults. This is attributed to an underdeveloped adaptive immune system in infants, and to waning natural immunity combined with co-morbidities in the older adult. A clear understanding of the mechanisms of natural-acquired adaptive immunity to *S*. *pneumoniae* is essential to characterise why both the young and elderly are at high risk of disease and for the development of effective preventative strategies. Vaccines based on the polysaccharide capsule of *S*. *pneumoniae* are highly protective against the capsular serotypes included in the vaccine preparation [3–5], and protection correlates with the level of anti-capsular antibody responses. It has generally been assumed that the type-specific anti-capsular antibodies that can develop in response to colonisation or episodes of infection are also the main mechanism of natural adaptive immunity against IPD [6, 7]. However, there is little good evidence supporting the concept that levels of anti-capsular antibodies predict risk of IPD in unvaccinated individuals.

As well as causing symptomatic disease, *S*. *pneumoniae* asymptomatically colonises the nasopharynx, affecting at least fifty percent of infants and approximately ten percent of adults [8]. Colonisation is an immunising event. In humans, it leads to antibody responses to capsular polysaccharide [9], but also induces both antibody [10–14] and cellular immune responses to protein antigens [15, 16]. Serum levels of antibody to multiple pneumococcal surface proteins rise in the first few years of life [13], and have been show to fall in older age for a limited number of antigens [17]. Similar adaptive immune responses are observed in mouse models of nasopharyngeal colonisation [11, 18–25]. In animal models, these anti-protein responses alone can be protective, with T-cell mediated immunity preventing re-colonisation and non-invasive pneumonia[15, 24, 25] and anti-protein antibody responses protecting against IPD [19, 20, 22, 24]. Recent human data suggests that Th17-cell mediated responses to protein antigens also play an important role in protection against colonisation in humans [26] with implications for vaccine design [27]. There are several converging lines of evidence from human studies which support the concept that naturally-acquired anti-protein antibodies can also protect against *S*. *pneumoniae* infections. Lower serum IgG levels to a range of pneumococcal proteins correlate with susceptibility to acute otitis media [28, 29] and respiratory tract infections in children [30]. Passive transfer of human serum from experimentally challenged human volunteers protected mice against invasive challenge with a different capsular serotype of pneumococcus [20], providing proof of concept that ‘natural’ antibodies against bacterial proteins induced through nasopharyngeal exposure can protect against IPD. Furthermore, the incidence of IPD falls after infancy for all serotypes of *S*. *pneumoniae*, irrespective of how commonly the serotype is carried in the nasopharynx [31] suggesting that naturally-induced adaptive immune mechanisms are serotype-independent. If the protection against IPD that develops naturally through colonisation requires anti-protein antibody responses rather than serotype-specific anti-capsular antibody, this would represent an important readjustment in our understanding of immunity to *S*. *pneumoniae*. It would have major implications for identifying subjects with an increased risk of infection, understanding mechanisms of immunosenescence that increase susceptibility to *S*. *pneumoniae* with age, and for guiding future vaccine design.

Passive transfer of pooled human immune globulin (IVIG) is an established treatment to prevent infections in individuals with primary antibody deficiency [32, 33], in whom *S*. *pneumoniae* is a leading cause of disease [34]. Previous investigations in mice have indicated that IVIG may protect against experimental IPD [35, 36]. Commercially-manufactured IVIG is pooled immunoglobulin G (IgG) from >1000 different donors [37], and therefore represents the pooled antibody responses acquired through natural exposure across a population. We have used IVIG to determine the targets of natural acquired immunity to *S*. *pneumoniae* and the relative functional importance of anti-capsular and anti-protein responses for prevention of IPD.

## Results

### IVIG contains IgG that recognises both *S*. *pneumoniae* capsular and proteins antigens

**Fig 1:**
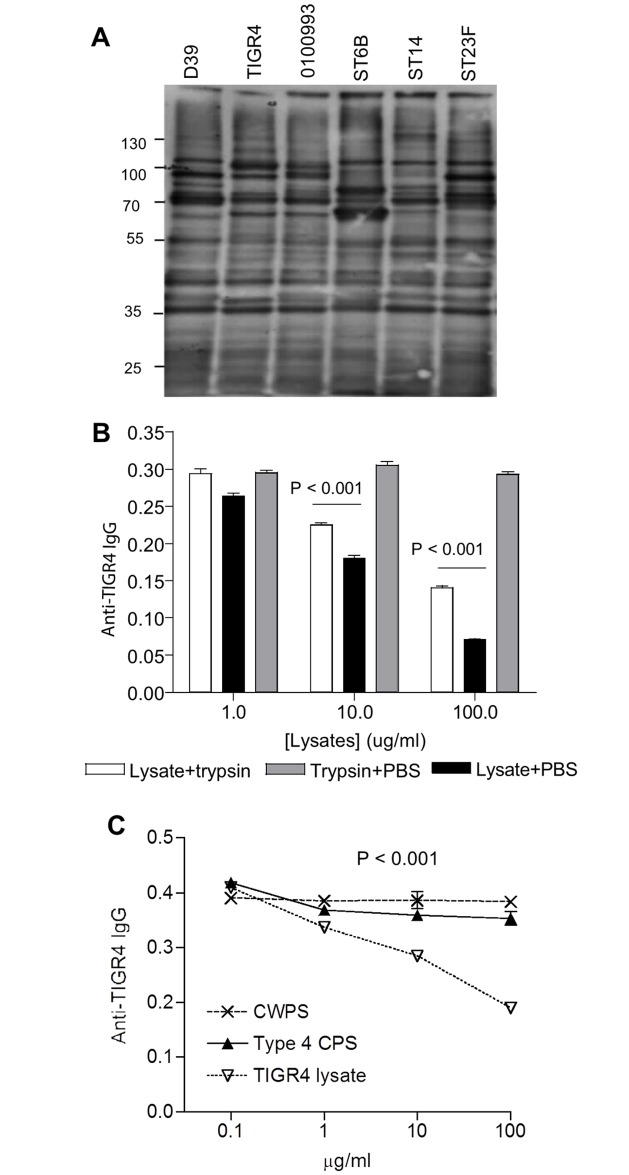
Antigen targets for IgG in IVIG. (**A**) Immunoblots of IVIG (1/1000) binding to whole cell lysates (10 μg / lane) of *S*. *pneumoniae* strains D39, TIGR4, 0100993, ST6B, ST14 and ST23F. (**B**) and (**C**) Competitive inhibition in IVIG (1/10000) whole cell ELISAs of IgG binding to solid-phase *S*. *pneumoniae* TIGR4 using increasing concentrations of (B) soluble lysates of TIGR4 with or without pre-treatment with trypsin, or (C) soluble purified cell wall polysaccharide (CWPS), soluble serotype 4 capsule (CPS), or soluble *S*. *pneumoniae* TIGR4 lysates. Data presented as means and SDs of four technical replicates and are representative of experiments repeated twice. *P* values calculated using 2-way ANOVAs and Bonferroni post-test comparisons; error bars represent SD.

**Fig 2:**
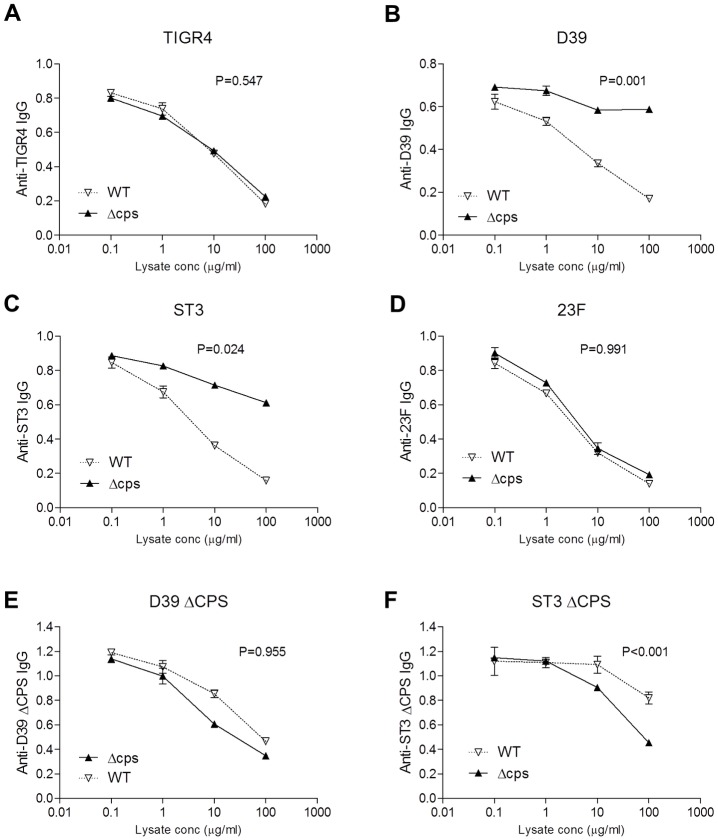
Effects of unencapsulated and encapsulated *S*. *pneumoniae* on IVIG whole cell ELISA titres for four strains. Competitive inhibition of IVIG (1/10000) whole cell ELISAs of IgG binding to the homologous solid-phase *S*. *pneumoniae* strain using increasing concentrations of encapsulated or unencapsulated bacterial lysates for wild-type **(A)** TIGR4, **(B)** D39, **(C)** serotype 3, (**D)** serotype 23F strains, and for unencapsulated mutant derivative of **(E)** serotype 3 and **(F)** D39 strains. Data presented as means and SDs of three technical replicates.

ELISAs using the whole *S*. *pneumoniae* cell as the antigenic target confirmed that IVIG contained significant titres of IgG that recognised *S*. *pneumoniae* (Table 1). Polysaccharide-specific ELISAs demonstrated that IVIG contained IgG that recognised common *S*. *pneumoniae* capsular serotypes and cell wall polysaccharide (CWPS) (Table 1). To assess whether IVIG contained IgG that bound *S*. *pneumoniae* proteins, immunoblots were performed against lysates of several *S*. *pneumoniae* strains of differing capsular serotypes. Multiple protein antigens were recognised by IVIG with a largely similar pattern of bands for all strains, suggesting the major protein targets of IVIG are generally conserved between capsular serotypes of *S*. *pneumoniae* (Fig 1A). Competitive inhibition was used to assess which antigens contributed significantly towards the whole cell ELISA titres for the TIGR4 strain. Pre-incubation of IVIG with a soluble bacterial lysate reduced whole cell ELISA IgG titres in a dose-dependent manner, which was partially reversed by pre-treating the soluble lysate with the protease trypsin (Fig 1B). In contrast, neither purified capsular polysaccharide nor CWPS affected whole cell ELISA IgG titres (Fig 1C). The whole cell ELISA assays were repeated for four different *S*. *pneumoniae* serotypes with competitive inhibition by encapsulated and unencapsulated bacterial lysates (Fig 2A–2D). The results demonstrated that for two of four strains lysates of encapsulated and unencapsulated bacteria equally reduced the IgG binding titre in the whole cell ELISAs. For the D39 (serotype 2) and serotype 3 strain whole cell ELISA titres were inhibited to a greater extent by lysates of encapsulated bacteria compared to unencapsulated bacteria. Further whole cell ELISAs for these two strains demonstrated that the unencapsulated mutants blocked IgG binding to unencapsulated mutants (Fig 2E and 2F), indicating the reduced inhibition in the whole cell ELISAs against the wild-type strain is likely to be due to the effects of anti-capsular antibody. These data show that IVIG contains antibodies to both capsular, CWP and protein antigens, but which class of antigens made the dominant contribution to IVIG recognition varied to an extent between *S*. *pneumoniae* strains when assessed using whole cell ELISAs.

**Table 1:** Antigen-specific IgG in IVIG. Whole cell ELISA titres to 3 different *S*. *pneumoniae* strains and quantity of capsular-specific IgG for 10 *S*. *pneumoniae* capsular polysaccharides as measured by multiplex MSD in IVIG (Intratect).

**Whole cell ELISA**	
**Strain (capsular serotype)**	**Whole cell ELISA titre (log10 at OD 0.1)**
TIGR4 (serotype 4)	4.72
D39 (serotype 2)	4.60
EF3030 (serotype 19F)	4.40
**Anti-capsular antibody level**	
**Serotype**	**IgG(ng/ml)**
1	22.3
4	10.4
5	23.4
6B	57.5
7F	33.1
9V	25.4
14	133
18C	36.4
19F	41.0
23F	40.2

### Identification of *S*. *pneumoniae* protein binding targets for human IgG

**Fig 3:**
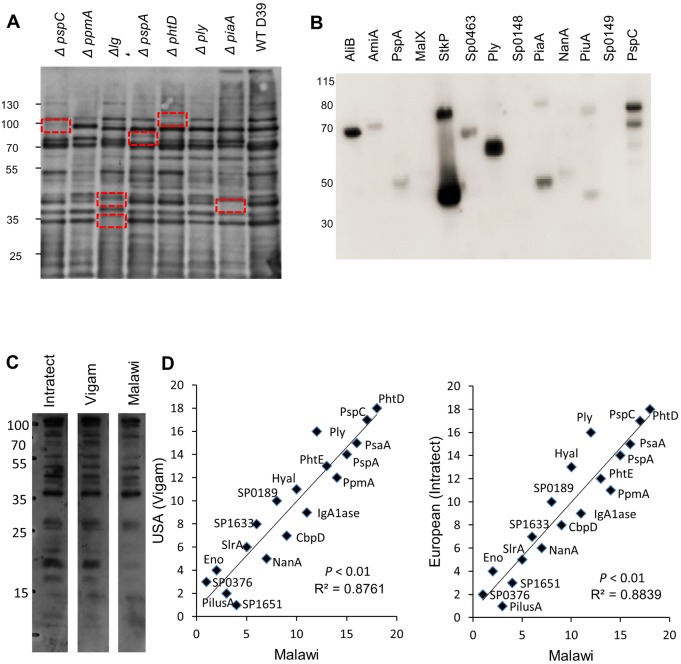
Identification of protein antigens recognised by different sources of pooled IgG. (**A**) Immunoblots of IgG (1/1000) binding to the wild-type *S*. *pneumoniae* strain D39 and isogenic mutant strains (10 μg) lacking specific surface proteins using IVIG. Boxes highlight missing bands corresponding to the molecular weight for the protein(s) absent in the mutant strains. (**B**) Immunoblots of IgG binding to selected recombinant *S*. *pneumoniae* proteins (0.5 μg / lane) probed with IVIG (1/500). (**C**) Immunoblots of IgG binding to wild-type *S*. *pneumoniae* D39 strain (10 μg / lane) using pooled sera from different geographical regions, commercial IVIG (1/3300) from Europe (Intratect) or USA (Vigam) and from Malawi. (**D**) Linear regression of the rank order of strength of IgG binding to different protein antigens between serum pooled from donors from Malawi and IVIG preparations obtained from the USA (Vigam) or Europe (Intratect). Data points represent the rank order of each protein antigen for the mean MFI of two technical duplicates for IgG binding measured using the Luminex assay. *P* and R^2^ values were obtained using F tests.

To identify protein targets for IgG in IVIG, lysates of *S*. *pneumoniae* mutants lacking specific surface proteins were probed with IVIG. The results showed that IgG in IVIG recognised the cell wall proteins PspA, PspC and PhtD and at least two lipoproteins (shown using the lipoprotein deficient strain *Δlgt*), including PiaA (Fig 3A). Immunoblotting of recombinant proteins confirmed that IVIG contains IgG that recognises multiple (but not all) *S*. *pneumoniae* protein antigens tested (Fig 3B). To assess whether protein targets for naturally acquired IgG to *S*. *pneumoniae* were conserved between donors from different geographical regions we performed immunoblots against *S*. *pneumoniae* lysates with a further commercially available IVIG preparation (Vigam) obtained from the USA, and with sera pooled from 20 Malawian subjects. The results showing an almost identical band pattern for each source of IgG (Fig 3C), suggesting a high degree of consistency for the major protein antigen targets for IgG obtained from different geographical regions. A Luminex assay of antibody binding to 19 different *S*. *pneumoniae* surface proteins conjugated to xMAP beads was used to semi-quantify responses from different sources of pooled human IgG to specific protein antigens. The Luminex assay confirmed that IgG in IVIG recognised multiple protein antigens including PsaA, PpmA, PhtD, PhtE, PspA, pneumolysin (Ply) and PspC (Fig 3D). Overall, the strength of IgG binding to individual *S*. *pneumoniae* protein antigens between the different sources of antibody correlated strongly, with PhtD and PspC as the dominant antigens in all three sources of pooled human IgG (Fig 3D, and for correlation of Vigram versus Intratech R^2^ = 0.966).

### *S*. *pneumoniae* target antigens vary partially between individuals and with age

**Fig 4:**
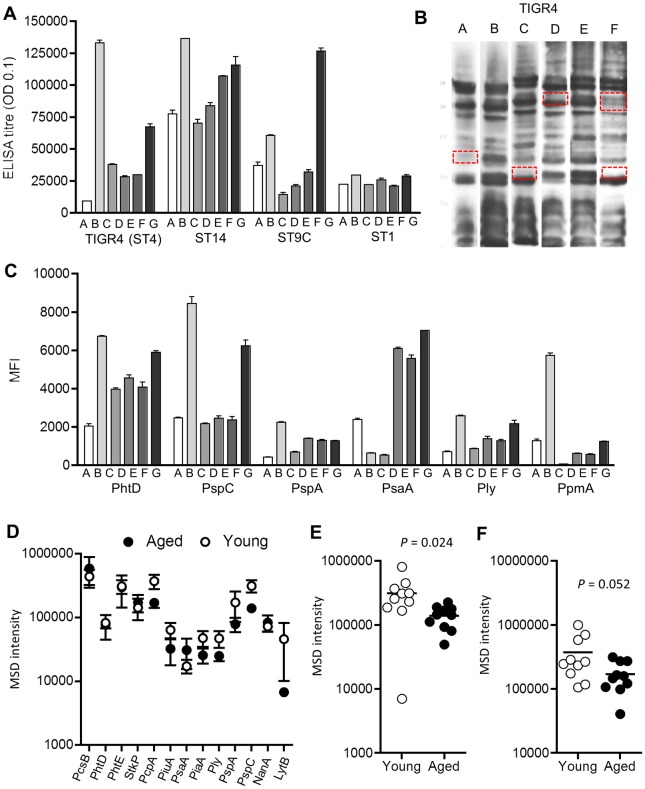
Variation of IgG antigen targets between individuals and with age. **(A-C)** Results for six Malawian subjects (labelled A to G). **(A)** whole cell ELISAs to 4 *S*. *pneumoniae* strains (serotype 4, 14, 9C and 1). **(B)** Immunoblots of IgG binding to the wild-type *S*. *pneumoniae* strain D39 (10 μg / lane, red boxes highlight bands that visibly vary in intensity between subjects). **(C)** MFI of IgG binding to selected protein antigens measured using a Luminex assay. Data points represent mean (SEMs) of two technical duplicates for IgG binding. For immunoblots and the Luminex assay, sera were diluted to 1/1000. **(D)** Levels of serum IgG binding to specific protein antigens aged (mean age 67.2 years, black symbols) and young subjects (mean age 31.2 years, empty symbols) measured by multiplex MSD (only results for antigens with stronger responses are shown as means and SEM). **(E-F)** Levels of serum IgG binding for each individual aged (black symbols) and young (empty symbols) adult subjects to the protein antigens PspC (E) and PcpA (F) measured by MSD. *P* values were calculated using unpaired T tests, with bars representing means for the group.

To assess whether there is significant variation between individuals in which *S*. *pneumoniae* antigens are recognised by naturally acquired IgG, whole cell ELISAs to four *S*. *pneumoniae* serotypes, immunoblots against *S*. *pneumoniae* lysates, the Luminex assay of protein antigen responses, and capsular serotype antibody ELISAs were repeated using sera from six young adult HIV negative Malawian individuals (mean age 29 years, range 21 to 36, 3 male, 3 female). The results showed all the individuals investigated have significant anti-protein antibody responses (Fig 4). However, there were variations between individuals in whole cell ELISA titres to different *S*. *pneumoniae* strains (Fig 4A) and the levels of antibodies to some protein antigens as shown by variations in band strengths in the immunoblot (Fig 4B) and in the results for the Luminex bead assay (Fig 4C). For all the strains tested whole cell ELISA titres from individuals correlated with the mean anti-protein antigen responses, whereas there was no correlation to anti-capsule antibody levels except for the serotype 1 strain (S1 Fig). These data support the hypothesis that anti-protein responses dominate IgG recognition of *S*. *pneumoniae* in human sera.

To investigate whether anti-protein antigen responses could be affected by age, an electrochemiluminescence-based multiplex assay based on MesoScale Discovery (MSD, Rockville, MD, USA) technology [13] was used to measure responses to 27 protein antigens in sera from 10 individuals aged over 62 years (mean 67.2 years) and 10 young adult individuals (mean age 31.2 years). In general, mean anti-protein antigen responses were slightly lower for the aged subjects (Fig 3D), with the most marked differences being for PspC (Fig 3E) and PcpA (Fig 3F). The difference between older and younger sera reached statistical significance for PspC.

### Human IgG binds to surface proteins on intact *S*. *pneumoniae* rather than capsular polysaccharide

**Fig 5:**
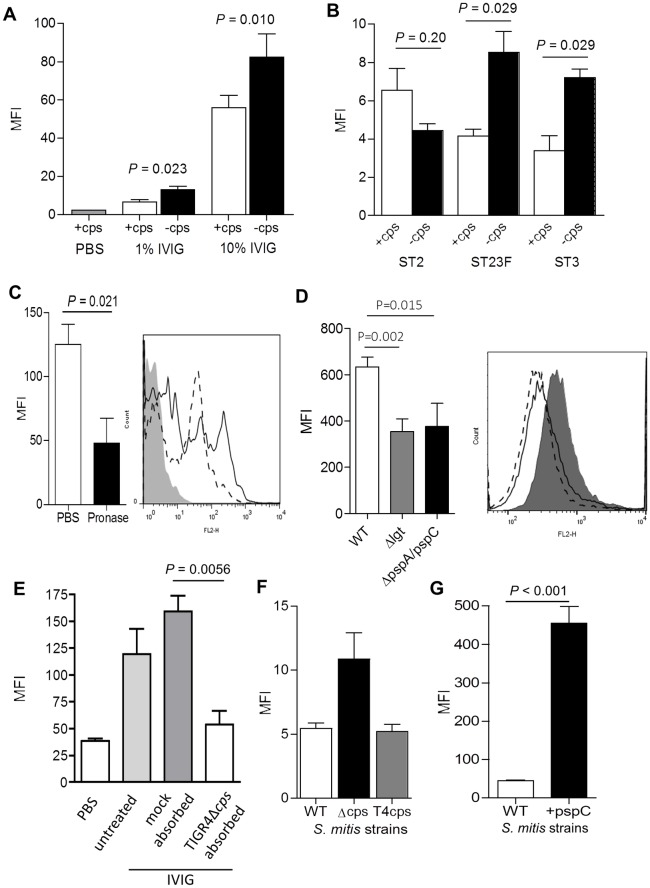
IgG binding to live *S*. *pneumoniae* after incubation with IVIG. (**A**) Effect of presence (wild-type strain, +cps) or absence (unencapsulated strain, -cps) of the capsule on IgG binding to live *S*. *pneumoniae* TIGR4 incubated in either 1% IVIG, 10% IVIG or PBS (control). IgG binding was measured as MFI using anti-IgG-PE by flow cytometry. (**B**) Effect of presence or absence of capsule on IgG binding to 3 other serotype strains (ST2, strain D39; ST23F; ST3, strain 0100993) incubated in 10% IVIG. (**C**) Effect of pre-treatment of bacteria with Pronase or PBS prior to incubation in 10% IVIG on IgG binding to TIGR4, and an example of the histogram for IgG binding MFI (solid line, PBS then IVIG; dotted line PBS then Pronase then IVIG; shaded area, no IVIG). **(D)** Effect of loss of expression of lipoproteins (*Δlgt*) or both the choline binding proteins PspA and PspC (*ΔpspA/pspC*) on IgG binding to the D39 strain after incubation in 10% IVIG, with an example of the histogram for the MFI of IgG binding (solid line, *Δlgt*; dotted line, *ΔpspA/pspC*; shaded area, D39). **(E)** Effect of depletion of specific surface protein antibodies from 10% IVIG using absorption with unencapsulated TIGR4 prior to incubating wild type TIGR4 bacteria in IVIG and measuring IgG binding using flow cytometry. **(F)** and **(G)** Effect of expressing potential *S*. *pneumoniae* antigens in *S*. *mitis* on IgG binding to live bacteria after incubation in 10% IVIG. (**F**) IgG binding to wild-type *S*. *mitis* (WT), *S*. *mitis* manipulated to lacking its own capsule (Δcps) or expressing *S*. *pneumoniae* serotype 4 capsule (T4cps). (**G**) IgG binding to wild-type *S*. *mitis* (WT) or *S*. *mitis* expressing PspC. For all panels, data are presented as means and SDs of three to four technical replicates and are representative of experiments repeated at least twice. *P* values were calculated using unpaired 2-tailed Student t-tests.

Functionally important IgG responses to *S*. *pneumoniae* were assessed using a flow cytometry assay to measure total IgG binding to intact live bacteria from different *S*. *pneumoniae* strains. Incubation in IVIG resulted in significant IgG binding to four different strains of *S*. *pneumoniae*. The level of IgG binding was either increased or unaffected when the assay was repeated using otherwise isogenic unencapsulated mutant derivatives of each strain, indicating that most of the IgG was binding to non-capsular antigens (Fig 5A and 5B). Conversely, pre-treatment with Pronase to degrade surface protein antigens (Fig 5C), using D39 mutant strains with reduced expression of dominant surface proteins (*Δlgt*, missing all lipoproteins, and *ΔpspA/pspC* missing the corresponding choline binding proteins) (Fig 5D), or pre-incubation of IVIG with an unencapsulated TIGR4 strain (Fig 5E), reduced the amount of IgG binding to the TIGR4 strain suggesting proteins were the target antigens. To further demonstrate that capsular polysaccharide was not the target for IgG binding, the assay was repeated using *Streptococcus mitis* strains genetically manipulated to express the serotype 4 *S*. *pneumoniae* capsule [37]. There was some binding of IgG in IVIG to the surface of the *S*. *mitis* strain indicating the presence of antibodies to surface antigens. However, there was no increase in IgG binding to the *S*. *mitis* strain expressing the *S*. *pneumoniae* serotype 4 capsule compared to wild-type *S*. *mitis* (Fig 5F). Conversely, expression by *S*. *mitis pspC*, one of the dominant *S*. *pneumoniae* protein antigens recognised by IgG in IVIG (Figs 3B, 3D, 4C and 4D), resulted in a large increase in IgG binding (Fig 5G). These results indicate that protein antigens (including lipoproteins, PspA and PspC) rather than capsular polysaccharide are the major surface targets for IgG binding to live *S*. *pneumoniae*.

### Enrichment for heterologous anti-protein antigen responses maintains protective efficacy of IgG from IVIG

**Fig 6:**
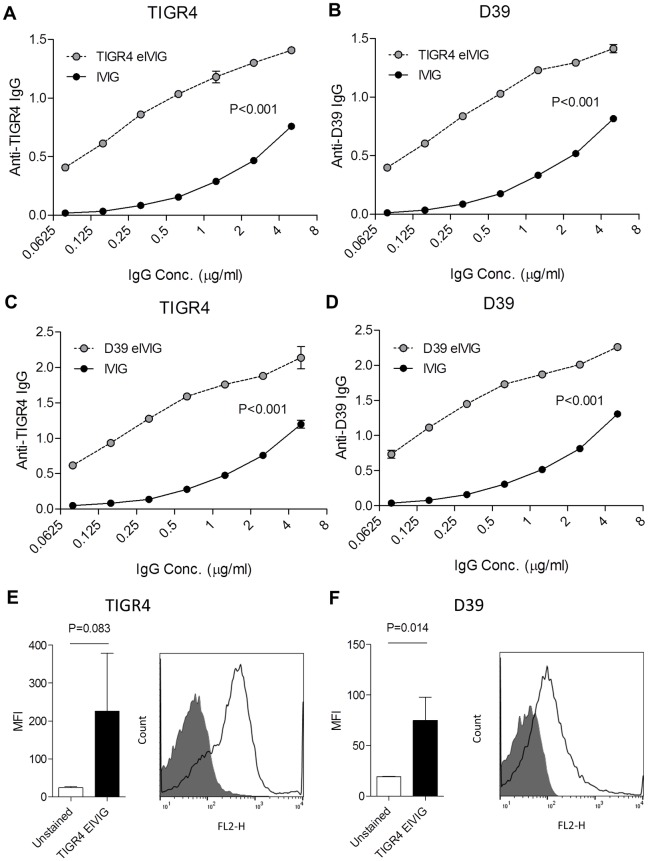
eIVIG immune recognition of *S*. *pneumoniae*. **(A-D)** Whole cell ELISAs of IgG binding to solid-phase *S*. *pneumoniae* of increasing concentrations of eIVIG made using TIGR4 **(A and B)** or D39 **(C** and **D)** or IVIG. Binding was assessed to the solid phase strain that was homologous (**A** and **D**) or heterologous (**B** and **C**) to the enrichment strain. (**E-F**) Bacterial surface IgG binding measured by flow cytometry to *S*. *pneumoniae* TIGR4 **(E)** or D39 **(F)** incubated in either PBS (shaded area) or TIGR4 eIVIG at 30 μg/ml (solid line). *P* values were calculated using linear regression for panels A, B, C, and D, and unpaired 2-tailed Student t-tests for panels E and F.

To further assess whether immune recognition of live *S*. *pneumoniae* is dependent on IgG recognition of protein antigens, IgG from IVIG was selectively enriched for responses to *S*. *pneumoniae* protein antigens using antibody affinity purification columns coated with unencapsulated *S*. *pneumoniae* lysates. The enriched IVIG (eIVIG) preparation made using either the TIGR4 or D39 unencapsulated strains had a markedly higher whole cell ELISA titres to both the TIGR4 and D39 encapsulated *S*. *pneumoniae* strains compared to untreated IVIG (Fig 6A–6D). Despite the eIVIG preparation IgG concentration being only 30 μg/ml, approximately 1/150 the concentration in IVIG, incubation in eIVIG still resulted in IgG binding to *S*. *pneumoniae* in the flow cytometry assay (Fig 6E–6F). These data confirm that IgG targeting *S*. *pneumoniae* protein antigens can mediate IVIG immune recognition of *S*. *pneumoniae*.

### IgG from IVIG promotes aggregation of *S*. *pneumoniae* independent of capsular antigen

**Fig 7:**
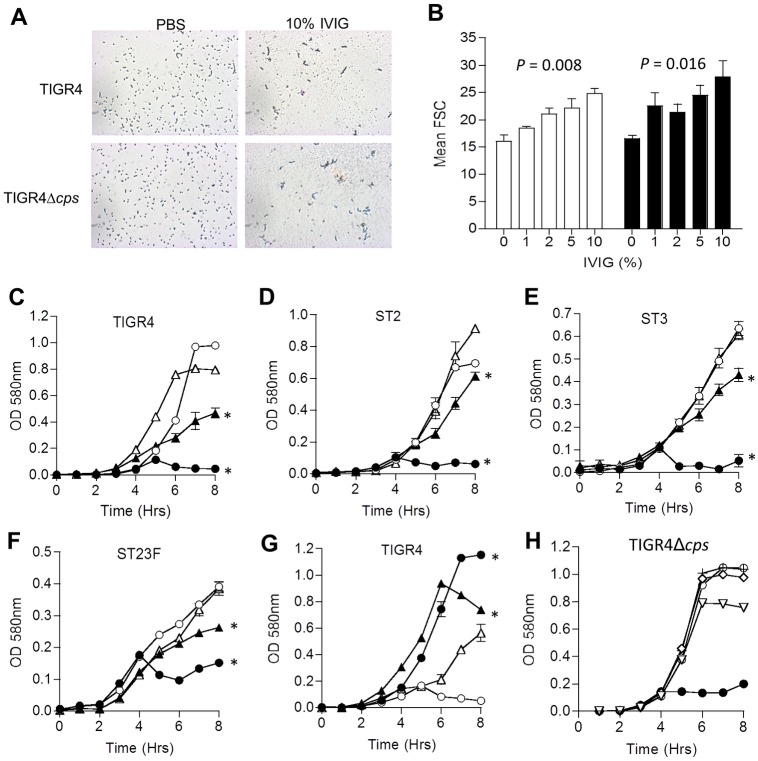
IVIG causes aggregation of both encapsulated and unencapsulated *S*. *pneumoniae*. (**A**) Light microscopy of bacteria (at 100 X with rapid Romanovsky stain) after 8 hr culture of wild-type (TIGR4) and unencapsulated (TIGR4Δcps) bacteria with or without 10% IVIG. **(B)** Effect of relative concentration of IVIG on size of bacterial aggregate particles after 30 minute incubation of wild-type (open bars) or unencapsulated (filled bars) TIGR4 measured as forward scatter in flow cytometry. *P* values were calculated using one-way ANOVA. **(C)** to **(F)** Changes in optical density at 580nm (OD_580_) during culture of wild-type (triangles) and unencapsulated (circles) *S*. *pneumoniae* **(C)** TIGR4, **(D)** ST2 (D39), **(E)** ST3 (0100993), **(F)** or ST23F strain in THY broth supplemented with either 10% IVIG (filled symbols) or PBS (empty symbols). **(G)** Effect of vigorous pipetting (filled symbols) or no pipetting (empty symbols) immediately prior to measurement of OD580 during culture of wild-type (triangles) and unencapsulated (circles) TIGR4 in 10% IVIG. **(H)** Effect of addition of either 1% IVIG (filled circles) or 10% (upside triangles), 5% (diamonds) or 2% (crosses) papain-treated IVIG, or PBS (empty circles) on OD_580_ during culture of unencapsulated TIGR4. For panels B to H, data are presented as means and error bars represent SDs of three to four technical replicates and are representative of experiments repeated at least twice. *, *P*<0.001 compared to PBS or non-pipetted controls by two way ANOVA.

IgG binding to *S*. *pneumoniae* can cross-link bacteria to form bacterial aggregates that are more susceptible to complement opsonisation [38]. Microscopy showed addition of IVIG to *S*. *pneumoniae* TIGR4 resulted in the formation of bacterial aggregates (Fig 7A), the relative size of which could be measured by flow cytometry using increases in forward scatter (Fig 7B). Both encapsulated and unencapsulated TIGR4 *S*. *pneumoniae* formed bacterial aggregates in IVIG, indicating these did not require recognition of capsular antigen (Fig 7A and 7B). Furthermore addition of IVIG restricted the increase in OD_580_ over time for different *S*. *pneumoniae* strains cultured in THY broth, and this effect was particularly noticeable for unencapsulated strains (Fig 7C–7F). Vigorous pipetting raised the OD_580_ to similar levels for both encapsulated and unencapsulated TIGR4 strains (Fig 7G), with no significant differences in numbers of bacterial CFU between the strains (log_10_ CFU / ml for the TIGR4 strain 7.60 SD 0.14, for the TIGR4Δ*cps* 7.85 SD 0.11 after 6 h incubation in THY plus 10% IVIG). These results indicated that the reduction in the increase in OD_580_ over time in THY containing IVIG was due to formation of bacterial aggregates. When IVIG was pre-treated with papain to yield monovalent Fab fragments, the majority of the inhibitory effect of increase in OD_580_ effect was lost, confirming that bacterial aggregation was caused by cross-linking of bacterial cells via the Fab portions of IVIG (Fig 7H). Overall, the aggregation data demonstrate that the dominant target antigen for functionally important IgG binding to *S*. *pneumoniae* incubated in IVIG was not the polysaccharide capsule.

### IgG from IVIG promotes phagocytosis of *S*. *pneumoniae* TIGR4 independent of capsular antigen

**Fig 8:**
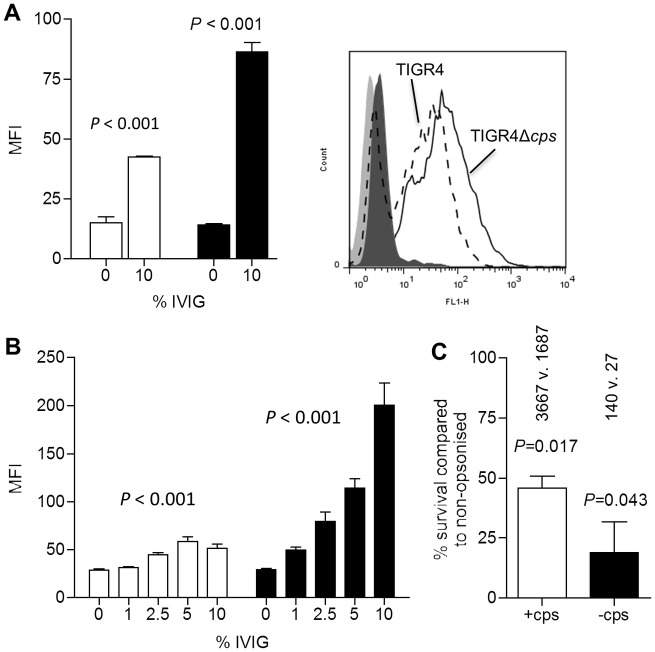
IVIG improves macrophage and neutrophil opsonophagocytosis of both encapsulated an unencapsulated *S*. *pneumoniae* TIGR4. (**A**) MFI and example of a histogram of the fluorescence of RAW macrophages incubated with fluorescent (FAM-SE labelled) encapsulated (clear columns) and unencapsulated (black columns) *S*. *pneumoniae* TIGR4 strains with and without addition of 10% IVIG. *P* values were calculated using unpaired 2-tailed Student t-test. **(B**) MFI of fluorescence intensity of human neutrophils following incubation with FAM-SE labelled *S*. *pneumoniae* TIGR4 encapsulated (clear columns) and unencapsulated (filled columns) strains pre-opsonised with increasing concentrations of IVIG. *P* values were calculated using one-way ANOVAs. (**C**) Relative survival of *S*. *pneumoniae* TIGR4 encapsulated (+cps) and unencapsulated (-cps) strains following incubation with human neutrophils in the presence of 10% IVIG. Percentage survival calculated relative to survival of identical strain incubated without IVIG, with the surviving CFU / ml shown for control versus 10% IVIG above each column. For all panels, data are presented as means and SDs of three to four technical replicates and are representative of experiments repeated at least twice. *P* values were calculated using unpaired 2-tailed Student t-test (**A** and **C**) or one way ANOVAs (**B**).

*In vitro* assays were used to assess the effects of IVIG on interactions of encapsulated and unencapsulated *S*. *pneumoniae* TIGR4 with phagocytes. Opsonisation with IVIG enhanced the association of *S*. *pneumoniae* with a murine macrophage cell lines (Fig 8A) and with fresh purified human neutrophils (Fig 8B), and enhanced neutrophil killing of *S*. *pneumoniae* (Fig 8C). For all three assays, IVIG had a proportionally greater effect on the unencapsulated strain than the encapsulated strain. These data support the hypothesis that anti-capsular IgG is not important in mediating the opsonophagocytic effects of natural human IgG present in IVIG.

### IVIG provides macrophage-dependent protection against TIGR 4 IPD in mice

**Fig 9:**
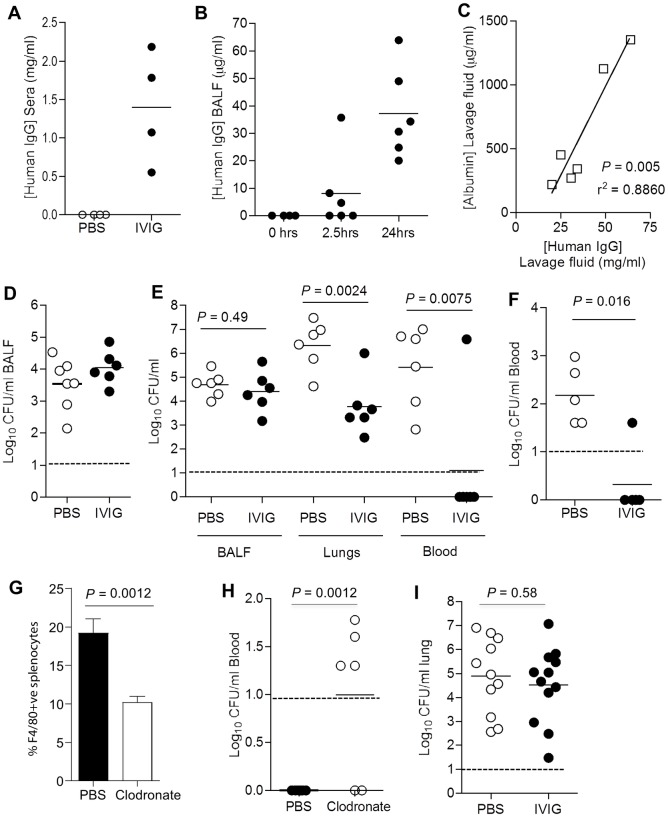
Passive vaccination with IVIG protects against IPD in murine models of *S*. *pneumoniae* infection. Mice were passively vaccinated by i.p. administration of 12.8 mg of IVIG (Intratect) or PBS 3 hours before challenge with *S*. *pneumoniae*. (**A**) and **(B)** Concentration of human IgG measured by ELISA after **(A)** in mouse sera 3 h post-IVIG (n = 4), and (**B**) in mouse BALF recovered from mice 3 h post-IVIG and immediately before and at 2.5 and 24 h after i.n. challenge with 10^7^ CFU of TIGR4 strain *S*. *pneumoniae* (n = 4 to 6). **(C)** Correlation between concentration of murine albumin (mg/ml) and human IgG (μg/ml) in BALF 24 hr following invasive i.n. challenge in IVIG-treated mice (n = 6); *P* and r^2^ values were calculated using the F test. **(D)** Bacterial CFU (log_10_) recovered from BALF 2.5 hr following IN challenge with 5x10^5^ CFU of TIGR4 of IVIG-treated or PBS-treated control mice (n = 6 or 7). **(E)** Bacterial CFU (log_10_) recovered from BALF, lung tissue or blood 24 hr following IN challenge with 10^7^ CFU of TIGR4 of IVIG-treated or PBS-treated control mice (n = 6). **(F)** Bacterial CFU (log_10_) recovered from blood 4 hr following i.v. challenge with 5x10^5^ CFU of TIGR4 of IVIG-treated or PBS-treated mice (n = 5). **(G)** Effect of administration of i.v. 100 μl liposomal clodronate (5mg/ml) to mice on the numbers of F4/80+ve splenocytes measured by flow cytometry (n = 6), with data presented as means, error bars represent SDs, and *P* values were calculated using unpaired 2-tail Student t-test. **(H)** Effect of clodronate or PBS administration on bacterial CFU (log_10_) recovered from the blood of IVIG-treated mice 4 hr following i.v. challenge with 5x10^5^ CFU of TIGR4 (n = 5). (**I)** Bacterial CFU (log_10_) recovered from the lungs of neutrophil depleted mice (by prior treatment with the antiLy6 antibody 1A8) given either IVIG or PBS 24 hr and then inoculated i.n. with 5x10^6^ CFU of TIGR4 (n = 11 or 12). For **(A, B, D, E, F, H, I)**, symbols represent data from individual mice, bars represent group means, and *P* values were calculated using unpaired 2-tail Student t-test. Dashed lines represent the limit of detection.

Passive vaccination was used to investigate the protective efficacy of IVIG in different murine models of *S*. *pneumoniae* TIGR4 infection. Mice were given a total of 12.8mg of IVIG (Intratect, Germany, 40 g/L) in two separate i.p. injections 3 h and immediately before challenge with *S*. *pneumoniae*. This IVIG dose was selected as it is equivalent to the doses used in replacement therapy in primary immunodeficiency. In a test dose experiment, human IgG was readily detectable in the sera of IVIG-treated mice three h following the second intraperitoneal injection at approximately 1.5 g/L, within the same order of magnitude of circulating IgG levels in humans (7+ g/L) (Fig 9A). Human IgG was not detectable in mouse bronchoalveolar lavage fluid (BALF) in uninfected mice. Following *S*. *pneumoniae* lung infection by i.n. inoculation of 5x10^6^ CFU of TIGR4, human IgG concentrations increased in BALF over time (Fig 9B) and correlated with BALF murine albumin levels, a marker of serum leak into alveolar spaces (Fig 9C). IVIG treatment had no effect on the inflammatory response to *S*. *pneumoniae* pneumonia, both in terms of inflammatory cell numbers (S2 Fig) or levels of the pro-inflammatory cytokine TNF-α in BALF post-infection (control group 2828 SEM 670 versus IVIG group 2665 SEM 506 pg/ml) in the lavage fluid following infection). IVIG treatment also had no effect on bacterial CFU in lavage fluid 2.5 hours after low dose inoculation with TIGR4 (Fig 9D), a time point and inoculum dose when alveolar macrophages are the main effector cell [38]. However, at 24 h following challenge, infected mice that had been pre-treated with IVIG were strongly protected against the development of bacteraemia (present in 100% of controls but only 17% of IVIG treated mice) and partially protected against lung infection, with 2 log_10_ fewer *S*. *pneumoniae* CFU in lung tissue compared to controls (Fig 9E). Pre-treatment with IVIG also protected mice against developing bacteraemia 4 h following direct i.v. bacterial challenge (Fig 9F). Protection against bacteraemia required macrophages, since their depletion by pre-treatment with liposomal clodronate (Fig 9G) reduced IVIG-dependent *S*. *pneumoniae* clearance from the blood (Fig 9H). The partial protection provided by IVIG within the lungs was lost when mice were depleted of neutrophils before infection by treatment with anti-Ly6G antibody (Fig 9I). Mice depleted of neutrophils failed to develop bacteraemia even without passive vaccination with IVIG. These data confirm that passive vaccination with IVIG strongly protects mice against IPD, and that protection was dependent on phagocytes.

### IVIG-mediated protection is not dependent on presence of anti-capsular IgG

**Fig 10:**
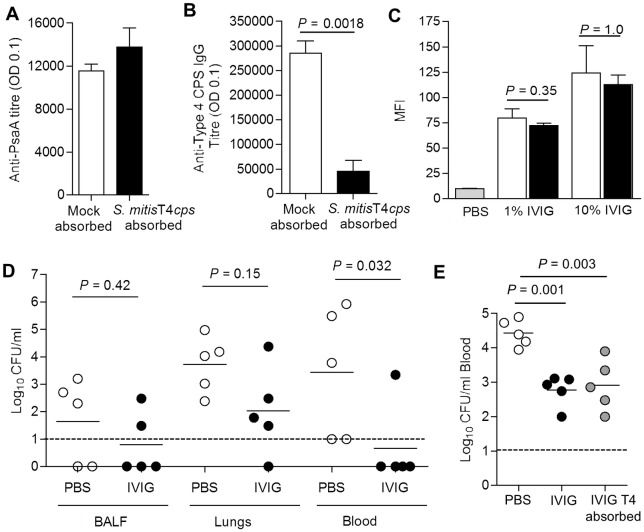
Absorption of anti-capsular antibody does not prevent IVIG protection against murine models of IPD. **(A)** Mean (SD) anti-PsaA titres measured by ELISA in IVIG following depletion of type 4 specific IgG by pre-absorption with type 4 capsule expressing *S*. *mitis*. (**B**) Mean (SD) anti-capsular serotype 4 titre IgG titre measured by ELISA in type 4 specific IgG-depleted IVIG. (**B**) Mean (SD) MFI of anti-human IgG binding to wild-type (clear columns) and unencapsulated (black columns) *S*. *pneumoniae* TIGR4 following incubation in 1% or 10% in type 4 specific IgG-depleted IVIG. (**C**) Bacterial surface IgG binding measured by flow cytometry to *S*. *pneumoniae* TIGR4 incubated in PBS (grey column), 1% or 10% mock absorbed IVIG with (filled columns) or type 4 specific IgG-depleted IVIG (open columns). For panels (A) to (C) data are presented as means and SDs of three to four technical replicates and representative of experiments repeated at least twice. (**D**) Log_10_ CFU recovered from BALF, lungs, and blood 24 h after i.n. challenge with 1x10^7^ CFU *S*. *pneumoniae* TIGR4 for mice pre-treated with 12.8 mg of type 4 specific IgG-depleted IVIG or PBS. (**E**) Log_10_ CFU recovered from blood 4 h after i.v. challenge with 5x10^5^ CFU *S*. *pneumoniae* TIGR4 for mice pre-treated with PBS (open circles), or 12.8 mg of mock absorbed IVIG (black circles) or type 4 specific IgG-depleted IVIG (grey circles). Data for panels **(D)** and **(E)** were obtained using IVIG depleted of capsular antibody on separate occasions; symbols represent data from individual mice, bars represent group means, and *P* values were calculated using unpaired 2-tail Student t-test **(D)** or one way ANOVA **(E).** Dashed lines represent the limit of detection.

To directly demonstrate that the protection afforded by IVIG is not mediated via anti-capsular antibody, IVIG was pre-treated to deplete anti-capsular antibody prior to testing its protective effects against IPD *in vivo*. Selective depletion of capsular serotype 4 specific antibody depletion was achieved by incubating IVIG with the *S*. *mitis* strain expressing the *S*. *pneumoniae* serotype 4 capsule. This process had no effect on the pattern and level of IgG binding to protein antigens in immunoblot and in ELISA for at least two specific proteins (Fig 10A). Whilst the depletion process almost completely removed serotype 4 anti-capsular IgG from the IVIG (Fig 10B), it had no effect on total IgG binding to the surface of *S*. *pneumoniae* when assessed by flow cytometry (Fig 10C). Passive transfer of IVIG depleted of type 4 serotype specific antibody to mice still protected against bacteraemia developing after i.n. inoculation of TIGR4 *S*. *pneumoniae* (Fig 10D), and after i.v. inoculation of TIGR4 restricted blood CFU to similar levels seen in mice given untreated IVIG (Fig 10E). These data confirm that IVIG does not require IgG to capsular polysaccharide to protect against invasive infection due to *S*. *pneumoniae*.

## Discussion

The bimodal distribution of *S*. *pneumoniae* infections in the very young and elderly suggests there is a significant degree of naturally-acquired immunity that evolves in early life and then wanes in later life. This naturally-acquired immunity is probably acquired through multiple episodes of nasopharyngeal colonisation with *S*. *pneumoniae* that repeatedly affect all humans rather than solely after disease episodes [16, 18–20, 24, 31]. Human epidemiological and experimental evidence from mouse models of infection suggest naturally-acquired immunity has a serotype-independent component [20, 28, 29, 31], yet the assumption remains that antibody to capsular antigen is the dominant mechanism of protection against IPD [6, 7]. As a consequence, clinical assessment of susceptibility to IPD is dependent on measuring anti-capsular IgG levels.

IVIG is a source of pooled IgG that contains naturally-acquired antibody to *S*. *pneumoniae*. We have used *in vitro* and *in vivo* experiments to compare the relative functional importance of the anti-capsular and anti-protein antigen IgG in mediating protection against *S*. *pneumoniae*. Overall, the data show greater importance for anti-protein rather than anti-capsular IgG, summarised as follows: (1) For both surface binding of IgG measured by flow cytometry and in vitro aggregation capsular antigen was not the main target for the four serotypes investigated. Data from the whole cell ELISAs were more mixed, with evidence of some contribution of anti-capsular IgG for two of the four strains assessed. However, IgG surface binding to live bacteria has been show to be a better surrogate for protection than ELISA titre [18]. (2) Enzymatic degradation of surface proteins, absorbtion of anti-protein antibody by incubation with unencapsulated TIGR4 strain, or reduced expression of some classes of surface proteins due to mutation all reduced total IgG binding to *S*. *pneumoniae*. (3) Expression by *S*. *mitis* of an immunodominant protein antigen but not the serotype 4 capsule increased IgG recognition when incubated in IVIG. (4) A low concentration of an IVIG derivative enriched for anti-protein responses to *S*. *pneumoniae* recognised heterologous *S*. *pneumoniae* strains in whole cell ELISA and flow cytometry IgG binding assays. (5) Loss of the capsule did not impair the protective effects of IgG in functional assays of neutrophil and macrophage phagocytosis of the TIGR4 *S*. *pneumoniae* strain. (6) Specific depletion of serotype 4 anti-capsular antibodies from IVIG had no effect on IgG binding to intact bacteria and did not abrogate the ability of IVIG to protect against IPD when tested in mouse models of infection. These data form the first evidence to our knowledge demonstrating the redundancy of naturally-acquired human IgG against capsular antigens in protection against IPD, with protection afforded by anti-protein antibody instead. By necessity, the four strains investigated represent only a proportion of the 97 *S*. *pneumoniae* capsular serotypes currently known [39], and we have only been able to deplete anti-capsular antibody for the serotype 4 strain as this is the only available *S*. *pneumoniae* capsular serotype expressed in *S*. *mitis*. Hence, although the in vitro aggregation and IgG binding data suggest capsule antigen is not functionally relevant for the four serotypes investigated, it remains possible that for selected serotypes anti-capsular antibody has a greater role in mediating protection against IPD than we have identified here. In addition, as we have not been able to make a sufficient quantity of an IVIG derivative effectively depleted of anti-protein antigen responses, we have not been able to explicitly demonstrate in the mouse model of infection that protection is dependent on anti-protein responses rather than to other potential non-capsule non-protein antigens. Our data also do not preclude an important role for naturally-acquired antibody to capsular antigens at other body sites, for example for prevention of nasopharyngeal colonisation [40]. Despite these caveats, the different strands of data we have presented here provide strong support for the hypothesis that the protection in humans against IPD mediated by naturally acquired IgG is not dependent on capsular antibodies. Instead protection seems to require recognition of bacterial surface proteins.

Protection against *S*. *pneumoniae* infection depends on phagocytes, with different cell types having dominant roles at different anatomical sites and at different time points. Alveolar macrophages are important for bacterial clearance during early lung function [38], whereas recruited neutrophils are important for controlling bacterial numbers in the lung at later time points [41]. In mice at least, protection against *S*. *pneumoniae* bacteraemia and therefore IPD is highly dependent on splenic and reticuloendothelial macrophages [42]. In the mouse model of *S*. *pneumoniae* lung infection, passive vaccination with IVIG did not reduce BALF CFU, even at early time points after low dose infection. These results suggest that alveolar macrophages did not mediate the protective effect, although this has not been formally confirmed by infections in mice depleted of alveolar macrophages. Depletion of neutrophils prevented the protective efficacy of IVIG within the lung, whereas systemic depletion of macrophages prevented its protective efficacy in the blood. Unexpectedly, depletion of neutrophils prevented septicaemia in the mouse model of pneumonia, preventing this model from being used to assess whether there is a role for neutrophils in IVIG-mediated protection against bacteraemia. Investigating this would require using neutrophil depletion in the systemic infection model, which we have not assessed. IVIG therapy has been used for immunomodulation, but in our model did not affect cellular recruitment to lavage fluid or TNFα responses. These results suggest that IVIG had no major effects on the inflammatory response to *S*. *pneumoniae*, although they do not exclude potentially beneficial effects on other aspects of the inflammatory response.

We have demonstrated that IgG in IVIG recognises a large number of *S*. *pneumoniae* protein antigens, several of which were identified using immunoblots and a Luminex assay and these include current protein vaccine candidate antigens [43]. There was a striking similarity between which protein targets were quantitatively dominant in binding IgG in IVIG from different geographical sources, with PspA, PhtD, PsaA and PpmA having the strongest antibody recognition in all IgG sources investigated. These similarities suggest that the immunodominance of certain protein antigens is largely independent of human genetic variation. Our protein target identification was biased towards existing well-described antigens, and further non-biased assessment is needed to identify all the antigens recognised by naturally acquired antibody. Several of the immunodominant surface proteins such as PspC and PspA are antigenically variable, and as only a single variant was represented on the Luminex assay it is unclear whether antibody recognition of these antigens is specific to certain alleles. Expression of PspC did increase IgG binding to live *S*. *mitis*, and for the D39 strain deletion of surface lipoproteins or both PspA and PspC both reduced IgG binding. These data suggest that PspC, PspA and lipoproteins may contribute towards IgG recognition of *S*. *pneumoniae*, but further investigation is necessary to identify which protein antigens are required for the protective IgG responses. This will be technically challenging as it is highly likely there is functional redundancy for IgG binding to *S*. *pneumoniae* surface proteins, and using mutants lacking specific protein antigens to identify functionally important targets for IgG in mouse infection models will be confounded by the importance for virulence of many of the potential protein antigens (e.g. PspA, PspC, Ply, PhtD). We also demonstrated IgG binding to *S*. *mitis* itself, which may be due to cross-recognition of *S*. *mitis* and *S*. *pneumoniae* surface antigens, or specific responses to *S*. *mitis* induced by natural oropharyngeal colonisation.

These data demonstrating that antibodies to *S*. *pneumoniae* capsular polysaccharide are not the major target of protective naturally acquired IgG have several important clinical implications. Firstly, measuring levels of anti-capsular antibody may not identify those patients at risk of IPD. Instead, measurement of antibodies to a range of protein targets or to whole *S*. *pneumoniae* by flow cytometry may be more relevant. Secondly, it may explain why individuals with specific-deficiencies in anti-polysaccharide antibody production, who are at increased risk of sino-pulmonary infection do not have the same high risk for invasive IPD as subjects with complete agammaglobulinaemia [44, 45]. Thirdly, the exponential rise in the incidence of *S*. *pneumoniae* infection with increasing age is thought to be related to immunosenescence. Antigen responses to a small number of protein antigens have been shown to be lower in the elderly [17], and we have also shown reduced responses to PspC in a small number of older subjects. These data suggest that one reason for the increased incidence of *S*. *pneumoniae* with age could be waning anti-protein antibody levels. Further investigation of the effects of age on anti-protein antigen responses and the functional consequences of any changes is needed to establish whether this hypothesis is correct. Fourthly, if there is reduction in *S*. *pneumoniae* colonisation in infants as a result of future vaccines with greater serotype coverage, this could potentially reduce anti-protein mediated natural immunity and perhaps lead to a paradoxical increase in adult disease, as has been postulated for the effects of *Bordetella pertussis* vaccination [46]. Finally, by identifying the mechanisms of naturally acquired immunity to *S*. *pneumoniae*, we can design vaccination strategies to improve these. For example, a multivalent protein vaccine using the dominant protein antigens should provide effective protection against IPD.

To conclude, we present multiple lines of supporting evidence that the protective benefits of human naturally acquired IgG against IPD is not, as previously thought, largely dependent on antibody to capsular polysaccharide antigen. Instead, natural human IgG-mediated protection against IPD seems to be dependent on IgG against protein antigens that are highly conserved between different geographical sources of IgG. These findings have important implications for identifying patients at risk of IPD, understanding relevant mechanisms of immunosenescence, and for novel vaccine development.

## Materials & Methods

### Bacterial strains, culture and manipulation

Wild-type *S*. *pneumoniae* serotype 4 strain TIGR4 and its unencapsulated mutant were kind gifts of J. Weiser (Univ. Pennsylvania). D39 and its unencapsulated mutant D39-DΔ were kind gifts of J. Paton (Univ. Adelaide). The Δ*pspC*, Δ*pspA*, Δ*ppmA*, Δ*lgt*, Δ*phtD*, Δ*piaA*, and Δ*ply* mutant strains have been previously described [47–51]. Serotype 19F strain EF3030 was a kind gift of D. Briles (Univ. Alabama), and the serotype 6B stain ST6B, serotype 14F strain ST14, and serotype 23F strains were kind gifts from B. Spratt (Imperial College). The unencapsulated mutant strains of 0100993 and ST23F were made by replacing the *cps* locus (Sp_0346 to Sp_0360) with the Janus cassette [52]. The *S*. *mitis* strain expressing the *S*. *pneumoniae* TIGR4 serotype 4 capsule has been previously reported [53]. To construct the *S*. *mitis* pspC+ mutant strain, the TIGR4 *pspC* gene was amplified by PCR and integrated between *S*. *mitis* flanking DNA using PCR ligation before transformation into the *S*. *mitis* strain, similar to the mutagenesis strategy as described [22]. Bacteria were cultured overnight at 37°C in 5% CO2 on Columbia agar (Oxoid) supplemented with 5% horse blood (TCS Biosciences). Working stocks were made by transferring one colony of *S*. *pneumoniae* to Todd-Hewitt broth supplemented with 0.5% yeast extract (THY), grown to an OD of 0.4 (approximately 10^8^ CFU/ml) and stored at -80°C in 10% glycerol as single use aliquots. CFU were confirmed by colony counting of log_10_ serial dilutions of bacteria cultured overnight on 5% Columbia blood agar. To partially digest surface proteins, bacteria were suspended in 500μl PBS with or without 100μg Pronase (Roche), incubated for 20min at 37°C shaking at 150rpm, followed by addition of 20μl of 25X Complete Mini-Protease Inhibitor (Roche). Bacteria were then washed twice in PBS and re-suspended in PBS+10% glycerol. Bacterial lysates were prepared as described previously [48]. When required, 20μl of lysate (1500 μg/ml) was treated with 10μl trypsin (2.5mg/ml, Gibco, Invitrogen) or PBS (control lysates) and incubated overnight, before the addition of 10μl 25X Complete Protease Inhibitor (Roche).

### Serum sources and IVIG

Intratect was a kind gift of Biotest Pharma GmbH, Dreieich, Germany. Vigam (Bioproducts Laboratories Ltd, Elstree, UK) was obtained commercially. Both contain 5% pooled human intravenous immunoglobulin. Dilutions of IVIG described for experimental data refer to dilutions of the 5% product rather than the resulting IgG concentration. Individual sera were collected from HIV-negative healthy adults in Malawi (age range 19 to 49 years, mean 29 years, 16 male and 4 female) who had not been immunised against *S*. *pneumoniae*. Serum from elderly subjects (age range 62 to 78 years, 6 males, 4 females) and young adult controls subjects (age range 24 to 33 years, 4 males, 6 females) was a kind gift from Dr Elizabeth Sapey, University of Birmingham. Specific antibody was depleted from IVIG by bacterial surface absorption with either unencapsulated TIGR4 or *S*. *mitis* expressing the serotype 4 capsule [53]. Bacteria were grown to OD_580_ 0.4, washed and re-suspended to OD 1.0 using PBS, and 4mls were pelleted by centrifugation before re-suspension in 1.8mls of IVIG (Intratect). The suspension was incubated for 1hr at 37°C, shaking at 100rpm. The antigen-depleted IVIG was recovered by centrifugation and the process repeated. Mock absorbed IVIG was prepared by following the same process but without addition of bacteria. IVIG was pre-treated with papain to yield monovalent Fab fragments using the Pierce Fab Preparation Kit according to the manufacturer’s instructions and confirmed by immunoblot. Enriched (e)IVIG was prepared by affinity chromatography as previously described [54]. For the affinity resin, unencapsulated TIGR4 or D39 cultures were grown for 16h, pelleted and resuspended in 1 volume of coupling buffer (0.1 M Sodium bicarbonate, 0.5 M Sodium chloride; pH 8.3). Cells were pressure lysed at 200 MPa using a pressure cell homogeniser (Stansted) and the resulting lysates were 0.2 μm filtered and dialysed against 5L of coupling buffer for 4 h at RT. Lysates were concentrated using Vivaspin 20 centrifugal concentrators with a molecular weight cut of 10 kDa (GE healthcare) and coupled to cyanogen bromide activated agarose (Sigma-Aldrich) at a concentration of approximately 1 mg/ml according to the manufacturer’s instructions.

### Serology and antibody assays

Whole cell, or specific antigen (individual proteins, capsular polysaccharide or cell wall polysaccharide) ELISAs were performed as previously [18, 55–57]. Recombinant PhtD was a kind gift of C. Durmort [58] and PsaA was a kind gift from J. Paton [59]. IgG binding to a panel of bacterial proteins and multiple capsular serotypes were assessed using Luminex assays [55] and electrochemiluminescence-based multiplex assay based on MesoScale Discovery (MSD, Rockville, MD, USA) technology as previously described [13, 55, 60]. For immunoblotting, bacterial lysates were separated by SDS-PAGE and transferred on to nitrocellulose membranes as previously described [36]. Membranes were probed with IVIG (Intratect) or pooled human sera (1:1000). To assess IgG binding to the bacterial surface, flow cytometry was performed as previously described [57, 61, 62].

### *In vitro* functional assays

Effects of IVIG on bacterial aggregation during growth were assessed by inoculating THY with 1x10^6^ of *S*. *pneumoniae* and measuing the OD_580_ over an 8 h period in the presence of 10% IVIG (Intratect, 40mg/ml IgG) or PBS. Following 8 h growth, cultures were fixed onto polylysine slides (VWR), stained with rapid Romanowsky staining (Diff-Quick) and imaged under light microscopy (Olympus, BX40) at 100X using Q capture pro software. Bacterial aggregation was directly assessed by incubating bacteria diluted in PBS to 1X10^6^ CFU/ml at 37°C in 5%CO_2_ for 1 hr with 0%, 1%, 5%, 10%, IVIG (Intratect 40mg/ml IgG). After fixation in 50μl 10% NBF, particle size was asessed by flow cytometry using a FACSCalibur with Cellquest and Flowjo software (BD Bioscience, UK) as a change in forward-scatter (FSC). Bacterial phagocytosis was measured as previously described as the association of FAM-SE labelled bacteria with either RAW 264.7 macrophages (MOI 10) [38, 53] or freshly isolated human neutrophils [57]. Briefly, RAW 264.7 murine cells were grown in RPMI supplemented with 10% heat-inactivated foetal calf serum. After washing, they were infected with FAM-SE labelled bacteria at an MOI of 10 which had been pre-incubated with IVIG or PBS for 30 mins at 37 C. After 45 min, cells were harvested with trypsin, fixed with paraformaldehyde (PFA) and fluorescence assessed using a FACS Calibur flow cytometer with Cellquest and Flowjo software (BD Bioscience, UK). For neutrophil phagocytosis, similarly opsonised labelled bacteria were incubated with freshly isolated human granulocytes for 30 min at MOI 20, after which they were fixed with PFA and assessed by flow cytometry. To assess bacterial killing by human neutrophils, pre-opsonised bacteria were incubated with freshly isolated granulocytes for 45 min after which they were serially diluted, plated and incubated overnight prior to colony counting.

### Murine infection models and assays

For passive immunisation experiments with IVIG, 6 to 8 week old age-matched outbred CD1 mice (Charles River, UK) received two i.p. injections of IVIG totalling 12.8mg IgG or the equivalent volume of PBS 3 h prior and immediately before *S*. *pneumoniae* TIGR4. Challenges were given either i.n. with 50μl of PBS containing 1x10^7^ CFU or i.v. with 100μl of PBS containing 5x10^5^ CFU. To ensure aspiration of the IN inoculum, mice were anaesthetised using 4% halothane (Vet-Tech). At the designated time points after inoculation, mice were culled and BALF, lung homogenates, and blood obtained for plating to calculate bacterial CFU as described previously [19, 48]. BALF was collected by instilling the lungs with 1ml PBS via an incision in the trachea. This was recovered by aspiration repeated three times. Splenic macrophages were depleted by i.v. administration of 100ul of 5mg/ml liposomal clodronate (controls were given PBS liposomes) [38, 63]. Macrophage depletion was confirmed by a 50% reduction in F4/80+ splenocytes by flow cytometry using anti-F4/80-phycoerythrin (Caltag). To deplete Ly6G+ neutrophils, 600 μg anti-Ly6G monoclonal antibody (1A8m, Bioxcell) was administered by i.p. injection 24 hours prior to infection challenge depletion, as previously [24], resulting in a 94.8% decrease in neutrophils recruited to lavage fluid 24 hours after infection. Murine albumin was measured by ELISA using a commercially available kit following manufacturer’s instructions (Bethyl Laboratories). Murine TNF-alpha was measured by ELISA and BALF cell counts in cytospins as previously described [24]. Human IgG was measured in murine samples using a commercially available ELISA kit following manufacturer’s instructions (Cambridge Bioscience).

### Statistics

Data are presented as group means with error bars representing standard deviations (SDs). Student’s unpaired T-test was used to compare the mean of two groups or analysis of variance (ANOVA) for comparisons between multiple groups, using Bonferroni post-test comparisons. F tests were used to assess if the slope of linear regressions were statistically different to 0. Statistical tests were performed using Graph Pad Prism software, and *P* values < 0.05 were considered significant.

### Study approval

Experiments were approved by the UCL Biological Services Ethical Committee and the UK Home Office (Project Licence PPL70/6510). Experiments were performed according to UK national guidelines for animal use and care, under UK Home Office licence. Blood samples were taken from human volunteers in Malawi with approval of the University of Malawi College of Medicine Research and Ethics Committee and the Liverpool School of Tropical Medicine Research Ethics Committee (Ref: 00.54).

## Supporting Information

S1 Fig Correlation between IgG binding in serum of individuals to total bacterial antigens (whole cell ELISA titres) with binding to either the homologous capsular polysaccharide (capsular ELISA titres, A-D) or with bacterial surface proteins (mean MFI for all protein antigens in the Luminex assay, E-H). R-squared values were obtained using F-tests.
(PPTX)

S2 Fig Cell counts in broncho-alveolar fluid 24 hours after challenge with 10^7^ CFU S. pneumoniae TIGR4 in IVIG-treated and PBS-control mice. (PPTX)
