# Correction: Naturally Acquired Human Immunity to Pneumococcus Is Dependent on Antibody to Protein Antigens

**DOI:** 10.1371/journal.ppat.1006259

**Published:** 2017-03-08

**Authors:** Robert Wilson, Jonathan M. Cohen, Mark Reglinski, Ricardo J. Jose, Win Yan Chan, Helina Marshall, Corné de Vogel, Stephen Gordon, David Goldblatt, Fernanda C. Petersen, Helen Baxendale, Jeremy S. Brown

There are errors in the penultimate sentence of the “*S*. *pneumonia* target antigens vary partially between individuals and with age” section in the Results. The citations to Fig 3D, Fig 3E and Fig 3F should refer to Fig 4D, Fig 4E and Fig 4F. The sentence should read: In general, mean anti-protein antigen responses were slightly lower for the aged subjects (Fig 4D), with the most marked differences being for PspC (Fig 4E) and PcpA (Fig 4F).
