# Supplementary material for: Naturally Acquired Human Immunity to Pneumococcus Is Dependent on Antibody to Protein Antigens
Source: PLoS Pathog. 2017 Jan 30;13(1):e1006137. doi: 10.1371/journal.ppat.1006137 (PMC5279798; doi:10.1371/journal.ppat.1006137)
Supplement: S1 Fig — R-squared values were obtained using F-tests. (PPTX) [file ppat.1006137.s001.pptx]

## Slide 1
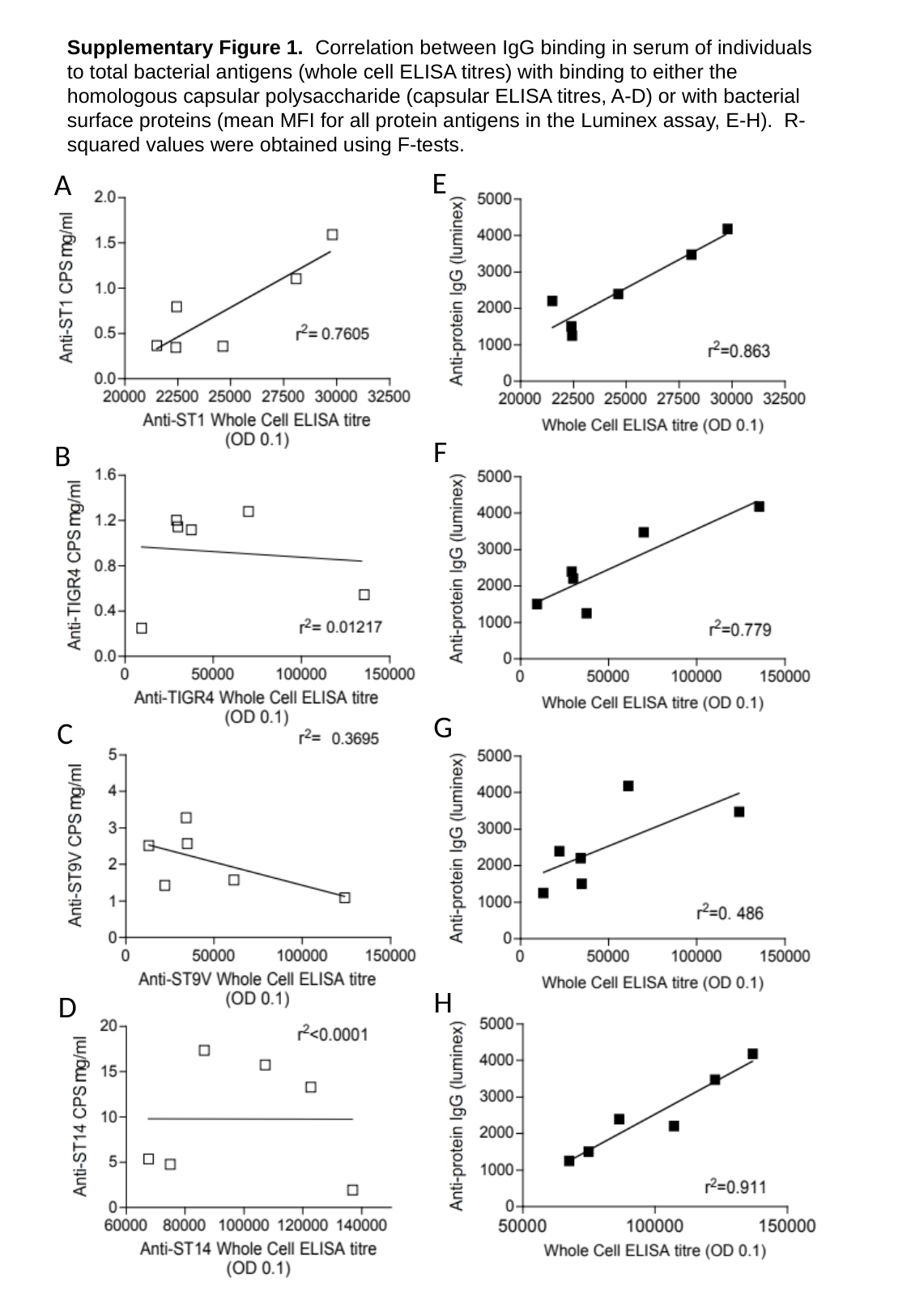

Supplementary Figure 1. Correlation between IgG binding in serum of individuals to total bacterial antigens (whole cell ELISA titres) with binding to either the homologous capsular polysaccharide (capsular ELISA titres, A-D) or with bacterial surface proteins (mean MFI for all protein antigens in the Luminex assay, E-H). R-squared values were obtained using F-tests.
E
A
F
B
G
C
H
D
