# Supplementary material for: Naturally Acquired Human Immunity to Pneumococcus Is Dependent on Antibody to Protein Antigens
Source: PLoS Pathog. 2017 Jan 30;13(1):e1006137. doi: 10.1371/journal.ppat.1006137 (PMC5279798; doi:10.1371/journal.ppat.1006137)
Supplement: S2 Fig — (PPTX) [file ppat.1006137.s002.pptx]

## Slide 1
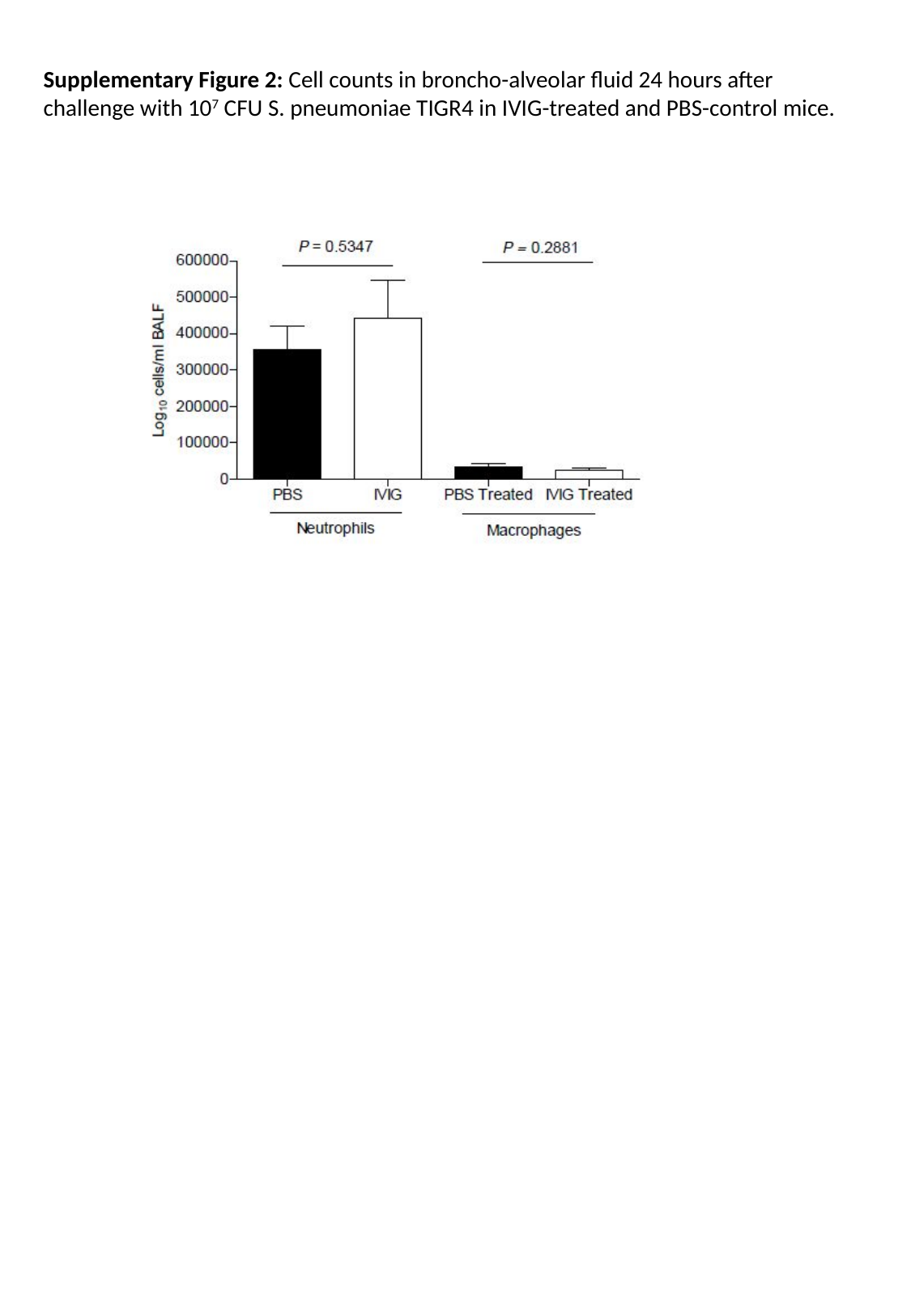

Supplementary Figure 2: Cell counts in broncho-alveolar fluid 24 hours after challenge with 107 CFU S. pneumoniae TIGR4 in IVIG-treated and PBS-control mice.
